# Androgens Regulate Gene Expression in Avian Skeletal Muscles

**DOI:** 10.1371/journal.pone.0051482

**Published:** 2012-12-17

**Authors:** Matthew J. Fuxjager, Julia Barske, Sienmi Du, Lainy B. Day, Barney A. Schlinger

**Affiliations:** 1 Department of Integrative Biology and Physiology, University of California Los Angeles, Los Angeles, California, United States of America; 2 Department of Ecology and Evolutionary Biology, University of California Los Angeles, Los Angeles, California, United States of America; 3 Laboratory of Neuroendocrinology, Brain Research Institute, University of California Los Angeles, Los Angeles, California, United States of America; 4 Department of Biology, University of Mississippi, University, Mississippi, United States of America; Universitat de Barcelona, Spain

## Abstract

Circulating androgens in adult reproductively active male vertebrates influence a diversity of organ systems and thus are considered costly. Recently, we obtained evidence that androgen receptors (AR) are expressed in several skeletal muscles of three passeriform birds, the golden-collared manakin (*Manacus vitellinus*), zebra finch (*Taenopygia guttata*), and ochre-bellied flycatcher (*Mionectes oleagieus*). Because skeletal muscles that control wing movement make up the bulk of a bird’s body mass, evidence for widespread effects of androgen action on these muscles would greatly expand the functional impact of androgens beyond their well-characterized effects on relatively discrete targets throughout the avian body. To investigate this issue, we use quantitative PCR (qPCR) to determine if androgens alter gene mRNA expression patterns in wing musculature of wild golden-collared manakins and captive zebra finches. In manakins, the androgen testosterone (T) up-regulated expression of parvalbumin (PV) and insulin-like growth factor I (IGF-I), two genes whose products enhance cellular Ca^2+^ cycling and hypertrophy of skeletal muscle fibers. In T-treated zebra finches, the anti-androgen flutamide blunted PV and IGF-I expression. These results suggest that certain transcriptional effects of androgen action via AR are conserved in passerine skeletal muscle tissue. When we examined wing muscles of manakins, zebra finches and ochre-bellied flycatchers, we found that expression of PV and IGF-I varied across species and in a manner consistent with a function for AR-dependent gene regulation. Together, these findings imply that androgens have the potential to act on avian muscle in a way that may enhance the physicality required for successful reproduction.

## Introduction

Androgenic hormones act on intracellular androgen receptors (AR) to influence local patterns of cellular gene expression. Because AR are expressed in diverse organ systems, androgens have pleiotropic effects on multiple physiological processes. As a consequence, when testicular release of testosterone (T) increases during the breeding season to activate masculine courtship and sexual behavior [1], [2], T might also enhance or diminish the function of target tissues that are adapted for optimal function in non-breeders [3]. However, much of the work that investigates how androgens affect various tissues does so by focusing on discrete androgen targets, including various brain nuclei [4], [5], components of the immune system [6], and reproductive organs [7], [8]. Significantly less is understood about how androgens influence relatively larger, more pervasive tissues and organ systems, particularly those that might impact day-to-day organismal performance [9], [10].

Skeletal muscles are a prominent tissue group within the vertebrate body, and in some species androgens may affect such tissue. Androgens appear to have robust effects on specific muscles that control movement important for reproduction, such as the musculature that controls penile reflexes [11], [12], mate-clasping behavior [13], [14], and courtship vocalizations [15]–[17]. In general, these muscles are relatively small and/or highly specialized for sex-specific behavior [18], [19]. However, a recent study in golden-collared manakins (*Manacus vitellinus*), zebra finches (*Taenopygia guttata*), and ochre-bellied flycatchers (*Mionectes oleagieus*) showed that AR are expressed in the main muscles that lift [*supracoracoideus* (SC) and *scapulohumeralis caudalis* (SH)] and retract [*pectoralis* (PEC)] the wings during flight [20]. This group of muscles make up nearly 40% of a bird’s body weight [21], [22], meaning that an enormous proportion of a bird’s mass is sensitive to androgen action. Consequently, the functional effects of androgen action in birds are likely far more widespread than previously thought, and this finding would have implications for the role that androgens play in mediating trade-offs in the transcriptional programs of various organ systems. To our knowledge, no studies have shown that androgens have functional (i.e. transcriptional) effects directly on avian skeletal muscle tissues that likely modulate muscle performance or morphology.

Given the information outlined above, the main goals of this study were to (*i*) measure the effects of androgens on gene expression patterns in avian wing muscles and (*ii*) assess the degree to which these effects are consistent among passerine species. To do this, we investigated four genes: parvalbumin (PV), insulin-like growth factor I (IGF-I), myogenic differentiation factor D (MyoD), and myostatin (MSTN). The products of these genes have well-characterized roles in skeletal muscle physiology and, to varying degrees, are influenced by androgen action in inbred rodent strains or cell cultures [23]–[25]. Additionally, some of these genes are thought to contribute to derived muscular phenotypes that underlie specialized behavior [26]–[28]. PV hastens muscle relaxation, and thus the contractile cycle of muscle fibers, by shuttling Ca^2+^ from troponin proteins within the muscle’s contractile machinery to the sarcoplasmic reticulum [29], [30]. IGF-I enhances muscle size and strength by inducing muscle cell proliferation and myotube hypertrophy [31], [32]. MyoD increases muscle strength and size by regulating the production of proteins that affect structure and fiber-type composition [33], [34]. MSTN negatively regulates muscle growth by arresting cellular division of myoblasts [35], [36].

We conducted three separate studies to address the issues outlined above, and in each of these studies we used quantitative PCR (qPCR) to measure mRNA expression levels in the main wing muscles that control avian wing movement (i.e, the SC, SH, and PEC). We predicted (*i*) that androgens up-regulate some of the genes of interest in passerine wing musculature and (*ii*) that these effects would be more pronounced in manakins. These predictions are based on past research showing that manakin wing muscles are generally larger and express more AR than those muscles of other similarly sized passerines [20], [37].

In our first study, we tested whether T treatment mediates mRNA expression of PV, IGF-I, MyoD, and MSTN in male manakins. In a subsequent study, we gave male zebra finches T implants and tested if the anti-androgen flutamide blocked T-induced expression of these same four genes. These two experiments allowed us to examine whether T influenced gene transcription in avian muscles similarly across species, and whether T did so through AR signaling. In a final study, we compared transcription of the target genes among reproductively active male golden-collared manakins, ochre-bellied flycatchers, and zebra finches. This final comparative study helped us to infer whether androgen-dependent gene expression is related to muscular AR levels, and whether constitutive expression of the target genes is consistent with each species’ predicted muscular needs. Ochre-bellied flycatchers served as a wild-living, suboscine control species with low levels of muscular AR, whereas the zebra finches served as a classic oscine model with similarly low levels of AR in skeletal muscles [20].

## Materials and Methods

### Animals

Experiments were conducted in accordance with all appropriate governmental agencies and approved by the Animal Care and Use Committees of the University of California, Los Angeles (UCLA), University of Mississippi, and the Smithsonian Tropical Research Institute (STRI). Male manakins were collected directly from their courtship leks, and flycatchers were collected from adjacent forest. Both species were caught using passive mist netting with nets inspected either constantly or at ∼30 min intervals. Reproductively active male zebra finches were taken from our colony at UCLA, where they were housed in a large (6′×6′×4′) same-sex, open-flying aviary. Males were in visual and acoustic contact with an adjacent aviary that contained reproductively active females.

### Androgen Modulation of Gene Expression in Manakins

We tested whether T treatment affects gene expression in wing muscles (SC, SH, PEC) of reproductively inactive male golden-collared manakins. Males used in this study were captured from late July to early September, as we have previously shown that T levels during this time of the year in males are virtually non-detectable [38]. Once males were transported to the research facilities at STRI laboratories, they were assigned at random to receive either a T implant (*n* = 3) or blank implant (*n* = 3) [20]. Implants were made from 12 mm of silastic tubing (0.76 mm i.d., 1.65 mm o.d.) that was filled with 10 mm of crystalline T (or nothing) and sealed at the ends with 1 mm of silastic adhesive. Implants were inserted subcutaneously at the base of the neck, and the incision made for insertion of each implant was glued back together with Vetbond Tissue Adhesive (3 M Animal Care Products, St. Paul, MN, USA). After implantation, birds were individually housed in small cages (32 cm×29 cm) and fed papaya *ad libitum* [39], [40]. Throughout the entire study, birds were kept in contact with at least one other individual, and separate cages were spaced no more than 5 cm apart. Prior studies have used this same method of T-treatment in manakins and shown that it increases circulating T to levels normally observed in reproductively active males captured at the onset of the breeding season, but does not impact overall health or locomotory behavior of the birds [39]–[41].

Birds were killed by rapid decapitation after 9–16 days of treatment. Whole bodies were immediately flash-frozen on dry ice. Carcasses were stored at −80°C and were later transported on dry ice to UCLA, where they were again stored at −80°C. Deep frozen muscle samples were dissected just prior to assays.

### Androgen Modulation of Gene Expression in Zebra Finches

We tested whether the AR antagonist flutamide suppresses T-induced gene expression in wing muscles of male zebra finches. First, birds were assigned at random to receive either an implant that contained flutamide (*n* = 6) or one that was empty (*n* = 6). On the following day, each bird received another implant that contained T. All implants were prepared in the same manner and with the same materials as described above. Implants were placed subcutaneously in fat pads of the zebra finches’ left and right flanks, immediately above the hip and below the wing. The incision into which implants were placed was glued back together with Vetbond Tissue Adhesive (3 M Animal Care Products). Birds were housed in small cages that were of a similar size to the cages used to house manakins. Each cage held three birds from the same treatment group. Prior studies show that this flutamide treatment is sufficient to block zebra finch AR without hindering an individual’s health or locomotory performance [42] and that this T treatment given to intact male zebra finches significantly increase plasma T within the species’ normal physiological range [43], [44].

All birds were killed by rapid decapitation 7 days after flutamide implantation. One flutamide-treated bird was eliminated from the analysis because the implant emerged from the initial incision halfway through the experiment. Muscle tissues were immediately collected and frozen on dry ice. Tissues were stored at −80°C until homogenization.

### Species Comparison of Gene Expression

Gene expression of the major wing muscles (SC, SH, PEC) was compared across reproductively active adult male golden-collared manakins (*n* = 4), ochre-bellied flycatchers (*n* = 4), and zebra finches (*n* = 6). Manakins and flycatchers were captured during their breeding season (January to July) and killed by rapid decapitation immediately upon removal from mist-nets. Carcasses or tissues dissected from carcasses were deep frozen in the field on dry ice and subsequently stored at −80°C. A visual inspection of the reproductive organs was performed to confirm that the gonads were enlarged and that the males were therefore in reproductive condition. At the end of the field season, carcasses and tissue samples were transported on dry ice to UCLA, where they were again stored at −80°C. Zebra finches were taken from their aviaries and killed in the same manner as the other species. Muscles were immediately dissected, frozen in dry ice, and stored at −80°C until homogenization. The reproductive organs were inspected and enlargement of the gonads was confirmed.

### PCR

Individual muscle samples were homogenized for 30 sec at medium speed with a rotor/stator homogenizer, and total RNA was isolated with Trizol® (Invitrogen, Carlsbad, CA) according to the manufacturer’s instructions. RNA concentration was determined with a Nanodrop system (Thermo Scientific, Wilmington, DE, USA), and RNA integrity was verified with gel electrophoresis.

Following DNase treatment (Promega, Madison, WI), 1 µg of RNA was reverse transcribed, using Superscript II Reverse Transcriptase (Invitrogen). This reaction occurred for 50 min at 42°C and then for 15 min at 70°C. The resulting cDNA was used for PCR amplification of PV, IGF-I, MyoD, and MSTN to confirm the presence of these genes in manakin, zebra finch, and flycatcher muscle tissues. PCR primers were created using annotated zebra finch sequences from the Ensembl database (Table 1). Each PCR reaction contained 0.38 mM of deoxynucleotide triphosphate, 0.4 µM of forward and 0.4 µM of reverse primer, 50 ng of cDNA, 0.06 ng of DNA taq polymerase (Bioline, Randolph, MA), and buffer. Reactions were run at 95°C for 5 min and then subjected to 39 cycles of 95°C for 30 sec, ∼64°C for 30 sec, 72°C for 1 min. Reactions were completed at 72°C for 10 min. PCR products were run on gel electrophoresis to ensure that the size of the actual product matched that which we expected, and a subset of products was sequenced (Genewiz Inc., La Jolla, CA, USA) and blasted against the zebra finch genome.

**Table 1:** PCR primers generated from zebra finch genome.

Gene	PrimerDirection	Sequence (5′-3′)	ProductSize (bp)
PV	Forward	TTTAATCTTTTCGCACTTGCTTC	338
	Reverse	CAATTTTACCATCACCGTCCTTA	
IGF-I	Forward	TCCTACATCCATTTCTTCTACCTTG	415
	Reverse	ACATTCATTCTTCATTCTTGTGGAT	
MyoD	Forward	GCAAGAGGAAGACCACCAAC	402
	Reverse	CGAGACTGGAAACAACAGAACTC	
MSTN	Forward	CTGAAAAGGACGGACTGTGC	1010
	Reverse	TACAACCATGGCTGGGATCT	

### Quantitative PCR

The procedures we used for qPCR are outlined in detail elsewhere [20], [41]. Briefly, all reactions were performed in an ABI 7300 sequence detection system, using SYBR Green Master Mix kits (Applied Biosystems Inc., Foster City, CA). Each reaction contained 5 ng of template, 0.9 mM of forward and 0.9 mM of reverse primers. Species-specific primers were developed for manakins, zebra finches, and flycatchers using product sequences obtained from the PCR results (Table 2). Primers for the internal control gene, glyceraldehyde-3-phosphate dehydrogenase (GAPDH), were based on annotated zebra finch sequence and used for all three species (Forward, 5′-TGACCTGCCGTCTGGAAAA; Reverse, 5′-CCATCAGCAGCAGCCTTCA, 70-bp product). GAPDH is a frequently used housekeeping control gene for qPCR studies; in general, its expression is unaffected by steroid treatment [45], [46], and our laboratory has verified that it does not detectibly differ in primer binding and reaction efficiencies among the examined species [20], [41]. All reactions were first run at 50°C for 2 min and then 95°C for 10 min. Each reaction was subsequently subjected to 40 cycles of 95°C for 15 sec and 60°C for 1 min. A dissociation stage was added to the end of the reaction process, consisting of 95°C for 15 sec, 60°C for 30 sec, and 95°C for 15 sec. Reaction efficiencies were between 90%–110%, and dissociation curves were used to verify the absence of contamination. Samples were run in duplicate. The standard curve method was used to measure the relative expression of each gene of interest (i.e., quantity gene of interest/quantity GAPDH).

**Table 2:** Species-specific quantitative PCR (qPCR) primers.

Species	Gene	Primer Direction	Sequence (5′-3′)	Product Size (bp)
Golden-collared Manakin	PV	Forward	GAAGGGCTTTACCCCAGAAG	72
		Reverse	TTATCTCCAGCAGCCAGAAG	
	IGF-I	Forward	AGGAGGCTGGAGATGTACTGTG	103
		Reverse	GCACTTCCTTTTGTGCTTTTG	
	MyoD	Forward	GGAATCACCAAATGATCCAAAG	74
		Reverse	CTACAATGCTTGAGAGGCAATC	
	MSTN	Forward	TGAACCCATTTTTAGAGGTCAG	85
		Reverse	GTGGAGTGCTCGTCACAGTC	
Zebra Finch	PV	Forward	TTGTCCTGAAGGGCTTTACC	93
		Reverse	TACCATCACCGTCCTTATCTCC	
	IGF-I	Forward	AACCAGTTCTGTTGCTGCTG	89
		Reverse	AAAGCCTCTGTCTCCACACAC	
	MyoD	Forward	ACAGAGTCCCCAAATGATCC	72
		Reverse	AATGCTTGAGAGGCAATCG	
	MSTN	Forward	ACTGCGTCTGGAACAAGCTC	195
		Reverse	AGAAAATCAGACTCTGTAGGCATTG	
Ochre-bellied Flycatcher	PV	Forward	AAGTTTGTCCTGAAGGGCTTTAC	100
		Reverse	TTTTACCATCACCGTCCTTATC	
	IGF-I	Forward	GGCTGGAGATGTATTGTGCTC	98
		Reverse	CACTTCCTTTTGTGCTTTTGG	
	MyoD	Forward	AGCAGGAGGACGCTTATTACC	110
		Reverse	AGGAGGCCCGCTGTATTC	
	MSTN	Forward	GCGATGGCTCTTTGGAAG	75
		Reverse	AATCAGACTCCGTAGGCATTG	

### Statistical Analysis

Analyses were performed using SPSS (v20.0). Data were natural log transformed [ln (1+X)] to meet the conditions of normality, and separate statistical models were used within each experiment to assess differences in mRNA levels for each gene. Species comparisons of gene expression were completed using a series of standard two-way ANOVAs, in which species and muscle were factors. Comparisons of gene expression in T-treated manakins were completed using two-way ANCOVAs, in which T-treatment and muscle were factors and implant duration was the covariate. Comparisons of gene expression in flutamide-treated zebra finches were completed using standard two-way ANOVAs, in which flutamide treatment and muscle were factors. Significant main effects found in ANOVA models were followed by Student-Newman-Keuls (SNK) post-hoc comparisons, and significant interactions were followed by simple main effect (SME) post-hoc tests. Significant main effects in ANCOVA models were followed by pairwise contrasts, with Bonferroni corrections applied to alpha values.

## Results

### PCR Amplification of Gene Products

We used PCR to confirm the presence of PV, IGF-I, MyoD and MSTN mRNA in skeletal muscle tissues of male golden-collared manakins, ochre-bellied flycatchers, and zebra finches. In all three species, PCR-amplification of these genes revealed bands of the expected size (PV: 338 bp; IGF-I: 415; MyoD: 402 bp; MSTN: 1010 bp).

Subsequent sequencing of the PCR products (Genewiz Inc., La Jolla, CA, USA) verified that the target genes were amplified. The sequence homology of each gene among the three species was relatively high (∼80% to ∼90%), and we used these sequences to develop species-specific primers for each gene of interest (Table 2). However, we used the same GAPDH (i.e., housekeeping control-gene) primer for all three species, because our laboratory has verified previously that there is no detectable difference in GAPDH mRNA expression or primer binding and reaction efficiencies among the separate species [20].

### Parvalbumin (PV)

**Figure 1:**
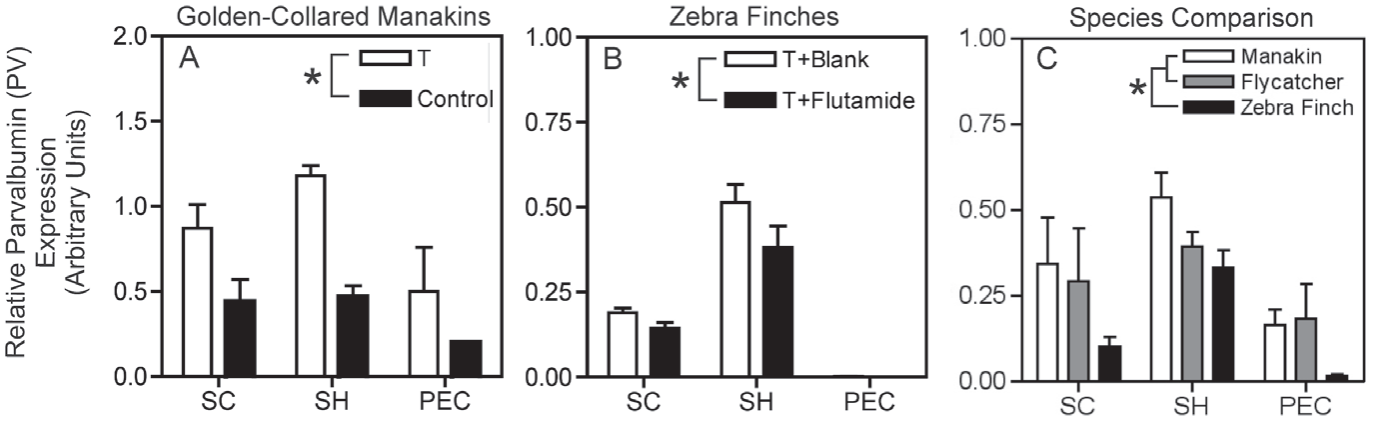
Relative expression of parvalbumin (PV) mRNA in the manakin testosterone (T) treatment study, zebra finch flutamide treatment study, and species comparison study. (A) Manakin T treatment study; white-bars represent male manakins treated with T-filled implants, and black bars represent male manakins treated with blank implants (Control). (B) Zebra finch flutamide study; white bars represent male zebra finches treated with T-filled implants and blank implants (T+Blank), and black bars represent male zebra finches treated with T-filled implants and flutamide-filled implants (T+Flutamide). PV expression in the PEC was virtually nonexistent compared to the other muscles and therefore is not clearly depicted on the graph. (C) Species comparison of reproductively active male birds. White bars represent golden-collared manakins, grey bars represent ochre-bellied flycatchers, and black bars represent zebra finches. Wing muscles are displayed on the horizontal axis in all graphs (*supracoracoideus* = SC; *scapulohumeralis caudalis* = SH; and *pectoralis* = PEC). Asterisks (*) displayed within each graph’s legend indicate significant differences between hormone manipulated groups (A & B) and among species (C). Data represent means ±1 SEM.

T treatment had robust, positive effects on PV mRNA expression in golden-collared manakins (Fig. 1A; *F_1,11_* = 41.61, *p*<0.001). Moreover, levels of PV mRNA varied across muscles (*F_2,11_* = 18.98, *p*<0.001), in that they were significantly higher in the SC and SH than in the PEC (SNK post-hoc, *p*<0.01). In manakins, PV expression was not influenced by a T*muscle interaction (*F_2,11_* = 3.54, *p* = 0.065).

Results in zebra finches were consistent with results in manakins, in that treatment with flutamide suppressed T-induced increases in muscular PV mRNA expression (Fig. 1B; *F_1,27_* = 4.37, *p* = 0.046). Again, PV mRNA levels differed across muscles (*F_2,27_* = 85.83, *p*<0.001); that is, PV expression was greatest in the SH compared to the SC and PEC (SNK post-hoc, *p*<0.05), though PV expression in the SC was significantly higher than in the PEC (SNK post-hoc, *p*<0.05). In effect, this means that PV mRNA was highest in the SH, intermediate in the SC, and lowest in the PEC. In zebra finches, PV was not influenced by a flutamide*muscle interaction (*F_2,27_* = 1.86, *p* = 0.18).

Among reproductively active males, we found that manakins and flycatchers expressed more PV mRNA than zebra finches in their wing muscles (Fig. 1C; *F_2,32_* = 5.77, *p* = 0.007; SNK post-hoc, *p*<0.05). PV mRNA also differed among muscles (*F_2,32_* = 11.77, *p*<0.001), whereby PV expression was greater in the SH than in the SC and PEC (SNK post-hoc, *p*<0.05) and greater in the SC than in the PEC (SNK post-hoc, p<0.05). Much like the result for T-treated manakins and zebra finches, PV mRNA was highest in the SH, intermediate in the SC, and lowest in the PEC in all three species. PV expression was not affected by a species*muscle interaction (*F_4,32_* = 0.36, *p* = 0.84).

### Insulin-like Growth Factor I (IGF-I)

**Figure 2:**
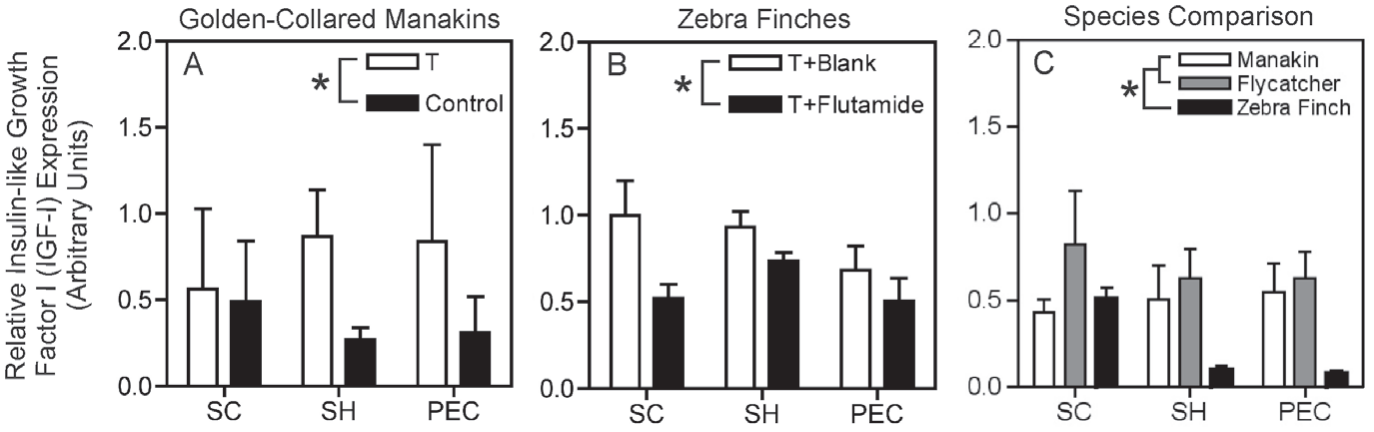
Relative expression of insulin-like growth factor I (IGF-I) mRNA in the manakin testosterone (T) treatment study, zebra finch flutamide treatment study, and species comparison stud. (A) Manakin T treatment study; white-bars represent male manakins treated with T-filled implants, and black bars represent male manakins treated with blank implants (Control). (B) Zebra finch flutamide study; white bars represent male zebra finches treated with T-filled implants and blank implants (T+Blank), and black bars represent male zebra finches treated with T-filled implants and flutamide-filled implants (T+Flutamide). (C) Species comparison of reproductively active male birds. White bars represent golden-collared manakins, grey bars represent ochre-bellied flycatchers, and black bars represent zebra finches. Wing muscles are displayed on the horizontal axis in all graphs (*supracoracoideus* = SC; *scapulohumeralis caudalis* = SH; and *pectoralis* = PEC). Asterisks (*) displayed within each graph’s legend indicate significant differences between hormone manipulated groups (A & B) and among species (C). Data represent means ±1 SEM.

In manakins, T administration increased IGF-I mRNA expression (Fig. 2A; *F_1,11_* = 8.36, *p* = 0.015). However, we found neither a difference in IGF-I mRNA among muscles (*F_2,11_* = 0.39, *p* = 0.96), nor a T*muscle interaction effect on IGF-I expression (*F_2,11_* = 1.06, *p* = 0.38).

As with the PV gene, the IGF-I data from zebra finches mirrored those IGF-I data from manakins; that is, flutamide treatment significantly blunted T-induced up-regulation of IGF-I mRNA production (Fig. 2B; *F_1,27_* = 6.93, *p* = 0.014). IGF-I mRNA did not differ across muscles (*F_2,27,_* = 1.74, *p* = 0.20) and was not affected by a flutamide*muscle interaction (*F_2,27_* = 0.81, *p* = 0.46).

Among reproductively active birds, manakins and flycatchers expressed more IGF-I than zebra finches in their wing muscles (Fig. 2C; *F_2,31_* = 8.64, *p* = 0.001; SNK post-hoc, *p*<0.05). There were no differences in IGF-I mRNA among muscles (*F_2,31_* = 1.49, *p* = 0.24), and there was no species*muscle interaction effect on IGF-I (*F_4,31_* = 1.13, *p* = 0.36).

### Myogenic Differentiation Factor D (MyoD)

**Figure 3:**
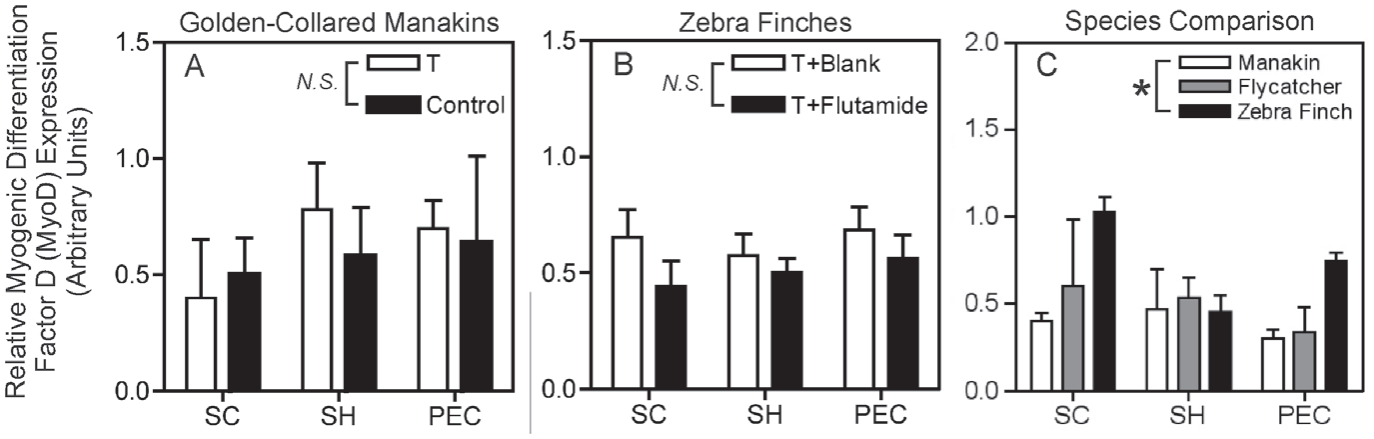
Relative expression of myogenic differentiation factor D (MyoD) mRNA in the manakin testosterone (T) treatment study, zebra finch flutamide treatment study, and the species comparison study. (A) Manakin T treatment study; white-bars represent male manakins treated with T-filled implants, and black bars represent male manakins treated with blank implants (Control). (B) Zebra finch flutamide study; white bars represent male zebra finches treated with T-filled implants and blank implants (T+Blank), and black bars represent male zebra finches treated with T-filled implants and flutamide-filled implants (T+Flutamide). (C) Species comparison of reproductively active male birds. White bars represent golden-collared manakins, grey bars represent ochre-bellied flycatchers, and black bars represent zebra finches. Wing muscles are displayed on the horizontal axis in all graphs (*supracoracoideus* = SC; *scapulohumeralis caudalis* = SH; and *pectoralis* = PEC). Non-significant differences between hormone manipulated groups (A & B) are denoted by the abbreviation *N.S.* within each graph’s legend, whereas significant differences among species (C) are denoted by an asterisks (*) displayed within the legend. Data represent means ±1 SEM.

We found no discernible effect of T treatment on MyoD mRNA expression in manakins (Fig. 3A; *F_1,11_* = 0.64, *p* = 0.44). Also, MyoD expression did not vary among muscles (*F_2,11_* = 2.35, *p* = 0.14) and was not affected by a T*muscle interaction (*F_2,11_* = 0.82, *p* = 0.47).

Once again, results in zebra finches paralleled results in manakins. Flutamide had no effect on MyoD mRNA in zebra finch muscles (Fig. 3B; *F_1,27_* = 2.65, *p* = 0.12), and MyoD expression did not vary among muscles (*F_2,27_* = 0.42, *p* = 0.66). There was no effect of a flutamide*muscle interaction on MyoD expression (*F_2,27_* = 0.26, *p* = 0.79).

Among reproductively active birds, zebra finches express more MyoD than manakins in the wing muscles (Fig. 3C; *F_2,32_* = 4.73, *p* = 0.016; SNK post-hoc, *p*<0.05), whereas MyoD expression in flycatchers did not differ from either manakins or zebra finches (SNK post-hoc, *p*>0.05). There was no difference in MyoD mRNA among muscles (*F_2,32_* = 1.78, *p* = 0.19) and no effect of a species*muscle interaction on MyoD expression (*F_4,32_* = 1.62, *p* = 0.20).

### Myostatin (MSTN)

**Figure 4:**
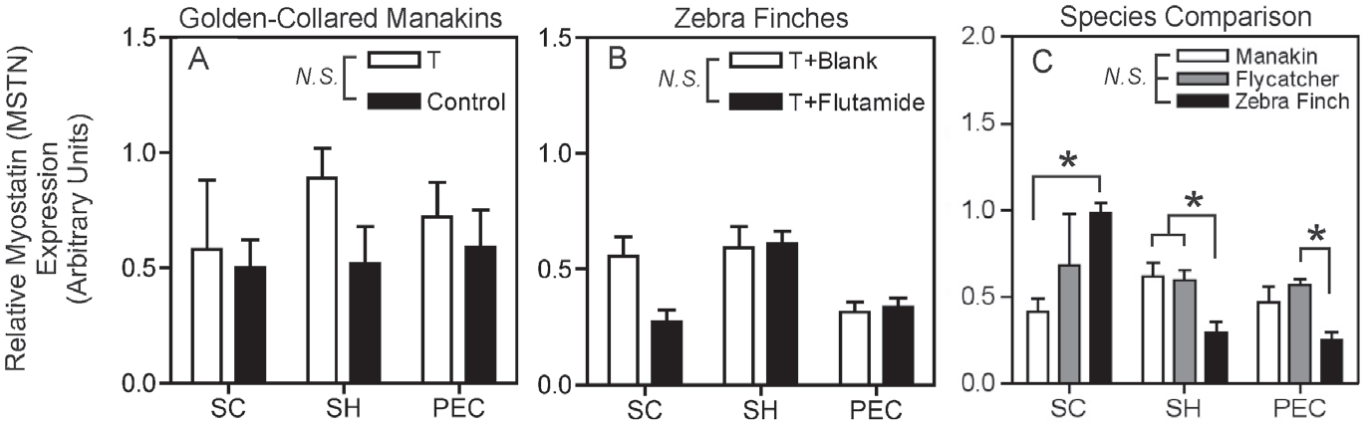
Relative expression of myostatin (MSTN) mRNA in the manakin testosterone (T) treatment study, zebra finch flutamide treatment study, and species comparison study. (A) Manakin T treatment study; white-bars represent male manakins treated with T-filled implants, and black bars represent male manakins treated with blank implants (Control). (B) Zebra finch flutamide study; white bars represent male zebra finches treated with T-filled implants and blank implants (T+Blank), and black bars represent male zebra finches treated with T-filled implants and flutamide-filled implants (T+Flutamide). (C) Species comparison of reproductively active male birds. White bars represent golden-collared manakins, grey bars represent ochre-bellied flycatchers, and black bars represent zebra finches. Wing muscles are displayed on the horizontal axis in all graphs (*supracoracoideus* = SC; *scapulohumeralis caudalis* = SH; and *pectoralis* = PEC). Non-significant differences between hormone-manipulated groups (A & B) and among species (C) are depicted by the abbreviation *N.S.* within each graph’s legend. Asterisks (*) displayed above the bars indicate significant differences between groups that we found following the detection of a significant interaction term. Data represent means ±1 SEM.

In manakins, we found no effect of T on MSTN mRNA levels (Fig. 4A; *F_1,11_* = 0.65, *p* = 0.44). There was also no difference in MSTN expression across muscles (*F_2,11_* = 1.43, *p* = 0.28) and no effect of a T*muscle interaction (*F_2,11_* = 1.29, *p* = 0.32).

We also found no discernible effect of flutamide on MSTN expression in zebra finches (Fig. 4B; *F_1,27_* = 2.31, *p* = 0.15). However, MSTN mRNA did vary significantly across muscles (*F_2,27_* = 8.78, *p* = 0.001), in that it was higher in the SH than the SC and PEC (SNK post-hoc, *p*<0.05). MSTN in these birds was not influenced by a flutamide*muscle interaction (*F_2,27_* = 3.30, *p* = 0.052).

Overall, we did not find an effect of species on MSTN expression (Fig. 4C; *F_2,32_* = 1.08, *p* = 0.35). However, MSTN expression did differ across muscles (*F_2,32_* = 4.99, *p* = 0.013), as it was generally expressed more in the SC than in the SH and PEC (SNK post-hoc, *p*<0.05). We also found that MSTN mRNA was significantly affected by a species*muscle interaction (*F_4,32_* = 6.10, *p* = 0.001), indicating that species differences in the expression of this gene were muscle-specific. Thus, manakins expressed more MSTN mRNA in the SC than zebra finches (SME post-hoc, *p*<0.01), while manakins and flycatchers expressed more MSTN mRNA in the SH than zebra finches (SME post-hoc, *p*<0.05). In the PEC, flycatchers expressed more MSTN mRNA than zebra finches (SME post-hoc, *p*<0.05).

## Discussion

Our results reveal that androgens are capable of modulating mRNA expression profiles of select genes in the wing musculature of two passerine species: golden-collared manakins and zebra finches. In general, androgens up-regulated the production of PV and IGF-I mRNA, with no detectable effect on the expression of MyoD or MSTN mRNA. Moreover, our results also showed that reproductively active males of these two species, as well as in males of a species of flycatcher, differed significantly with respect to constitutive expression of these genes. Taken together, these data not only identify AR-dependent effects of androgens on avian skeletal muscle [20], but also imply that such effects can influence the physiological performance of wing muscle tissues [29]–[32]. This work therefore expands our knowledge of where androgens can act within the avian body and thus lays the foundation for a conceptual framework in which we can consider the role of androgen-muscle interactions in various behavioral and physiological trade-offs.

### Avian Skeletal Muscle as an Androgen Target

To our knowledge, this study is among the first to demonstrate that androgens regulate gene transcription in avian skeletal muscle. These effects more than likely occurred in response to androgen activation of AR that is produced locally in the wing muscles. This idea is supported by studies showing that such tissues contain abundant 5α-reductase, which converts T into its more potent androgenic metabolite (i.e., dihydrotestosterone [47]), and relatively little estrogen receptor (ER), which binds the aromatized metabolite of T (i.e., estradiol) [20]. Other work has similarly detected high levels of AR and low levels of ER in avian muscle tissues, namely those muscles that control the syrinx, or avian vocal organ [15]. This past research led to the hypothesis that androgens act on AR to drive sex differences in syringeal size. However, the impact of these receptors in the syrinx is not completely clear, as flutamide administration in male zebra finches failed to change the fiber size of the two main syringeal muscles [48]. Our work does not examine the syrinx *per se*, but it suggests that AR in avian muscle is functional and thus further studies are needed to uncover the significance of syringeal AR.

Overall, our main finding that androgens modulate mRNA expression in wing muscles is especially significant considering the large body of research dedicated to uncovering the role of androgens in avian reproduction and life history trade-offs [49]–[52]. Within this body of work, studies predominantly consider the effects of androgens and AR on the nervous and immune systems [53]–[55]. However, wing muscles of passeriform birds comprise up to 40% of an individual’s total body weight [21], [22], which means that a significant proportion of a bird’s tissue mass is influenced by circulating androgens that fluctuate seasonally and as a result of social interactions [56]–[58]. Thus, transcriptional programs in wing muscles that are adaptive outside the breeding season (i.e., for increased flight) may be altered by androgens to facilitate reproductive behavior (see below), a potential cost to the males. It is important to note that such effects might also occur with no obvious change in either basal or peak metabolic rates [59], [60].

Evolutionarily, the result that androgens have significant effects on flight muscles also means that a relatively large portion of the passerine body is a substrate on which sexual selection can act to adjust the signaling properties of androgens and consequently modify (or even incorporate) body movement involved in sex behavior. Given these findings, it is perhaps no surprise that many avian species have evolved unique and elaborate courtship displays that require extraordinary and unusual motor control of the wings [61]–[63]. This, of course, implies that muscular androgen sensitivity may play an important role in the evolution of adaptive motor control, a hypothesis that merits further investigation.

### Importance of Androgen-induced Changes to Muscular Gene Expression

While prior studies have established that both golden-collared manakins and zebra finches express AR in the SC, SH, and PEC, this work also reveals that manakins express relatively more AR in these muscle tissues than zebra finches [20]. Here, we found that the overall positive effects of androgens on PV and IGF-I expression were relatively similar between these two species, regardless of their variation in muscular AR expression. This result implies that (*i*) even the low levels of AR detected in zebra finches wing muscles are functional [20] and that (*ii*) the ability of androgens to adjust select components of the transcriptional profiles of muscles, at least among passerine birds, is to some degree conserved. The latter point is supported taxonomically, because golden-collared manakins and zebra finches represent species from the suboscine and oscine suborders, respectively, that make up the larger avian order, Passeriformes [64]. However, despite these generally conserved effects, our data provide evidence that some variation in muscular transcriptional responsiveness to androgens may occur between species. PV, for example, is expressed at very low levels in the zebra finch PEC, and thus PV transcription in this muscle responds poorly to androgens, unlike its response in manakins. Further investigation of such species-level differences is warranted and is a focus of our laboratory.

We suspect that a driving force for the evolution of the physically demanding manakin courtship display involves the androgen-sensitivity of avian skeletal musculature. To attract females, males forcefully and rapidly flip their wings above their heads to produce a loud, firecracker-like wing-snap [38], [65]. This behavior is activated in part by AR [66] and ultimately used by females to pick mates, which suggests that sexual selection favors males with superior muscular strength and motor skills [67]. As such, expression of abundant AR in manakin wing muscle is thought to be a physiological adaptation to promote wing-snapping [20], and in this vein we expect that AR-dependent up-regulation of PV and IGF-I expression is one functional mechanism of this adaptation. In other words, circulating T likely elevates transcription of these two genes in a manner that coordinates optimal muscle performance with male display behavior. This idea is broadly supported by research showing that PV enhances the speed of the muscular contractile cycle [68] and that IGF-I increases the size and strength of muscle fibers [69]. Additional work is needed to elucidate the mechanisms through which these gene products might influence muscular performance of male courtship. One goal of our laboratory is to determine if manakin muscle morphology and physiology change seasonally, as this may provide insight into the gene networks involved in governing muscular adaptations.

### Androgen-independent Mechanisms of Muscular Gene Expression

In both manakins and zebra finches, neither MyoD nor MSTN were influenced by androgen manipulation. Prior studies that examine the effects of androgens on the expression of MyoD have given contradictory results [70], which may be due to differences in the examined tissue or muscle type [71]. Our results suggest MyoD is androgen-insensitive in passerine skeletal muscle. For MSTN, however, prior work leads to the expectation that androgens down-regulate its expression [72]. However, the research that would otherwise lead to this prediction comes from studies in mammals, and recent work suggests that the MSTN promoter differs greatly between mammals and birds [73]. Such differences may account for the seemingly differential effects that androgen action appears to have on the regulation of MSTN between these two vertebrate classes.

### Muscular Differences in Gene Expression

Our findings uncover that the transcriptional profile of certain genes vary among the wing muscles we examined. PV mRNA levels, for example, are relatively higher in the SC and SH than in the PEC. It is unlikely that these differences are due to variation in muscular androgen sensitivity, since AR is expressed in similar amounts across these muscle tissues in both species [20]. The notion that other mechanisms also contribute to constitutive differences in transcription of PV is further supported by the results in zebra finches, which indicate that PV expression is not completely abolished after blocking AR with flutamide. Additionally, MSTN expression appears to be higher in the SH than in the SC or PEC, though this result is only detected in zebra finches. The mechanisms that underlie this difference are not directly informed by this study, because MSTN itself does not appear to be regulated by AR (see above).

The significance of these differences in gene expression is currently unknown and difficult to deduce based on our study. It is, of course, possible that interspecific variation in the genetic phenotypes of flight muscles corresponds to species differences in wing-use or other flight needs [74], [75]. Such needs may not be related to the production of elaborate wing movements during courtship, but instead might affect other species-specific behavioral traits linked to survival or reproduction. Likewise, inter-muscle variation in gene expression might be owed to factors related to our experimental design (see below), again suggesting that functional significance of such variation should be considered with caution.

### Species Differences in Muscular Gene Expression Patterns

Among the three passerine species we examined, PV and IGF-I mRNA expression was greatest in the wing muscles of reproductively active male manakins and flycatchers. This result runs counter to our original prediction that AR-dependent gene expression would be higher in the manakin, as this species has greater muscle AR expression [20] and performs a substantially more “athletic” and physically demanding courtship display [76], [77]. In light of our findings, it is plausible that elevated PV and IGF-I is adaptive in this flycatcher; that is, elevated expression of these genes in wing muscles might enhance motor control of wing movement related to the modest – though not acrobatic – flutter-flight and hover-flight displays that males use to attract females [77]. This leads to the possibility that androgenic effects on skeletal muscle tissues are associated with wing displays used for sexual social interactions.

We cannot, however, dismiss the possibility that alternate factors drive the observed differences in constitutive expression patterns of PV and IGF-I. Such factors might include species differences in diet and activity, particularly because manakins and flycatchers were wild and zebra finches were from a captive population. Research in birds suggests that feeding patterns and bouts of exercise can impact mRNA levels of genes like MSTN and/or IGF-I in a host of peripheral tissues, including muscle [26], [78]. At the same time, we can determine that diet and activity are unlikely to confound the observed effects from our androgen manipulation experiments, because manakins and zebra finches within their respective studies were fed the same diet and housed in identical small cages that limited the physical activity of all birds equally. Moreover, prior work has shown that similar T treatments of manakins and flutamide treatments of zebra finches do not change overall activity levels or grossly impair or alter obvious locomotory performance [40], [42].

Despite the apparent androgen-*independent* nature of MyoD and MSTN, the expression of these two genes varied among species. MyoD was expressed more in zebra finches than in manakins, while there was no difference in expression of this gene between flycatchers and the other two species. We originally predicted that manakins would express more MyoD because the product of this gene leads to muscle hypertrophy [33], [34] and manakin wing muscles are generally larger than zebra finch wing muscles [37]. Similarly, MSTN expression varied among the three species in a manner that we did not predict; that is, manakins (and to some extent flycatchers) expressed less MSTN in the SC and more MSTN in the SH than zebra finches. We predicted that MSTN would be generally lower in manakin muscles, particularly the SH, because MSTN is a potent negative regulator of muscle growth [35], [36]. MyoD and MSTN may not be the primary regulators of muscle size in adult passerines. A broad transcriptomic analysis of muscular gene expression across species, muscle, sex and reproductive state may identify alternate genes regulating muscle size/function in manakins, and our lab is interested in conducting such an analysis.

### Conclusions

We show that (*i*) androgen action regulates the expression of various genes in the skeletal muscles of two passerines and that (*ii*) expression of these genes varies across bird species. To our knowledge, this is one of the first illustrations of androgen-dependent gene expression in avian skeletal muscles, and it greatly expands our understanding of where and how androgens can act in the bird body to impose “costs” on large tissue groups that make up a significant proportion of an individual’s wet mass.
